# The Organization of Self-Knowledge in Adolescence: Some Contributions Using the Repertory Grid Technique

**DOI:** 10.3390/ejihpe10010031

**Published:** 2020-02-05

**Authors:** Maria João Carapeto, Guillem Feixas

**Affiliations:** 1 Escola de Ciências Sociais, Departamento de Psicologia, Universidade de Évora, 7005-345 Évora, Portugal; mjoaocarapeto@gmail.com; 2 Facultat de Psicologia, Department of Clinical Psychology and Psychobiology, Universitat de Barcelona, 08035 Barcelona, Spain; 3 Institute of Neurosciences, Universitat de Barcelona, 08035 Barcelona, Spain

**Keywords:** self, identity, cognitive conflict, adolescence, personal constructs

## Abstract

(1) Background: This study aims to explore the usefulness of personal construct psychology as a comprehensive framework and assessment tool to embrace a diversity of self-knowledge organization constructs, and to account for developmental differences across adolescence. (2) Methods: The repertory grid technique was used to measure self-knowledge differentiation, polarization, discrepancies between Actual Self, Ideal Self, and Others, and implicative dilemmas, a particular kind of intrapersonal conflict. Data were collected from two samples of early and late adolescents, respectively. (3) Results: Globally, they showed that the organization of self-knowledge was different in both samples. In particular, older adolescents revealed a less polarized self-knowledge. In addition, they tended to construe higher Actual–Ideal self-discrepancies and to present more internal conflicts. No differences were found between early and late adolescents concerning global differentiation and the discrepancies between the self (Actual and Ideal) and the Others. (4) Conclusions: Despite the limitations of the study (e.g., small sample size, cross-sectional design), these novel results support the suitability of the repertory grid technique to capture developmental changes in self-knowledge organization during adolescence, as well as the explanatory potential of personal construct psychology to advance their understanding.

## 1. Introduction

Self-knowledge is a central issue in adolescent development [1,2,3]. Self-knowledge can be considered as a personal theory (or theories) that a person construes based on his/her experience, so as to be able to anticipate the events of his/her world, to inspire him/her with appropriate (adaptive) actions in each moment, as well as to maximize psychological well-being (e.g., [3,4,5,6]). The transformations of adolescence, like body changes (e.g., [7,8]), new social contexts and expectations [3,7,9], and cognitive advances in adolescent lives [10,11], press for changes in both the content and structure of self-knowledge [5]. Thus, adolescents can count on new guides to make sense of a diversity of events and inspire adaptive and autonomous behaviors in a multitude of situations.

Many constructs have been proposed to describe the structure and organization of self-knowledge in the context of adolescence by approaching, in different ways, the issues of differentiation, integration, and/or, less often, internal conflicts. This research line has provided important evidence and theoretical advances, but it still faces some challenges. First, studies that include several of these constructs of self-structure are scarce (except for self-esteem). Second, the constructs (and measures) utilized across the literature vary greatly, making them difficult to compare to each other, thus challenging or hampering researchers and practitioners’ efforts to obtain a comprehensive understanding of their role in the organization of self-knowledge (e.g., [12,13]). Third, some authors defend the superiority of idiographic data to assess self-structure, but measures based on (self-) descriptions provided by researchers prevail [12,14]. Finally, certain variables considered central in theory have been underrepresented in research, as it is the case of internal conflicts. Therefore, this study addresses some of these limitations by exploring self-knowledge organization in adolescence using George Kelly’s personal construct psychology (PCP) [6] as a comprehensive framework, both theoretically and methodologically (see also [15,16]).

### 1.1. The Perspective of Personal Construct Psychology on Self-Knowledge

From the beginning of life, a person lives immersed in a succession of events, objects, other people, as well as the experience of oneself. PCP [6] proposes that, based on the similarities and differences perceived between events, one builds bipolar dimensions of meaning (the personal constructs; for instance, “quiet–communicative”) and, in this way, makes life more predictable. The self and significant others are the most challenging and crucial elements to make sense of for an individual’s adaptation to the contexts, mainly social, we live in. So, construing self and others is the core psychological activity for adaptation, according to Kelly.

The self is, when considered in the appropriate context, a proper concept or construct. It refers to a group of events which are alike in a certain way and, in that same way, different from other events. The way in which the events are alike is the self. That also makes the self an individual, differentiated from other individuals [6] (p. 131).

The self is thus the pole of a “self–no-self” bipolar construct (or, even better, a “self–others” construct), “which in turn is construed” [16] (p. 456). Therefore, self-knowledge can be defined as a system of personal constructs conceived by the person from the similarities and differences they perceive between other people and themselves, and that is useful for the person to anticipate events and guide successful action in the interpersonal world (see also [17]). Thus, in this theory, knowledge about the self is entangled with knowledge about significant others, as proposed by other theories of the self [18,19] which has also been supported by cognitive neuroscience [20]. Furthermore, people construe a multiplicity of selves or a community of selves [21]. In addition to the Actual Self, people construe, for instance, an Ideal Self, selves as perceived by various others, or selves in particular contexts, relationships, or roles (see also [22]).

Moreover, Kelly’s theory addresses how these personal theories change according to the flow of experience. In order to prevail, personal constructs need to be validated against experience. When a personal construct system is not able to anticipate events adequately, invalidation occurs in a process that activates unpleasant emotional experience [16]. Then, some degree of revision of the current self-system is needed.

According to Kelly, constructs are not isolated units of meaning. Rather, they relate to other constructs in a hierarchical network where some constructs are superordinate and subsume others, the subordinate constructs. At the top of the system, core constructs are more stable and resistant to change than subordinate or peripheral constructs as they assure a sense of continuity and personal identity. Otherwise, their invalidation would render invalid large portions of the self-system and compromise a person’s ability to predict events. In addition, the structure of personal construct systems is proposed to increase in differentiation and hierarchical integration as life goes on, thus becoming more able and flexible in predicting a larger array of personal and interpersonal events.

In sum, in a certain sense, PCP also comprises a developmental point of view, although different from a normative developmental approach. Yet this perspective can be useful to understand change in self-knowledge organization in the context of daily life events and emotional experience. Despite often being regarded as a psychology of adults, PCP addresses human-construing processes in general and does not exclude children and adolescents. Indeed, in his masterwork, Kelly [6] profusely uses examples of a child’s construing. Jablonsky and Lester [23] noticed the scarcity of developmental studies (cross-sectional or longitudinal) in the PCP field, especially those concerning changes throughout adolescence and adulthood.

**Figure 1:**
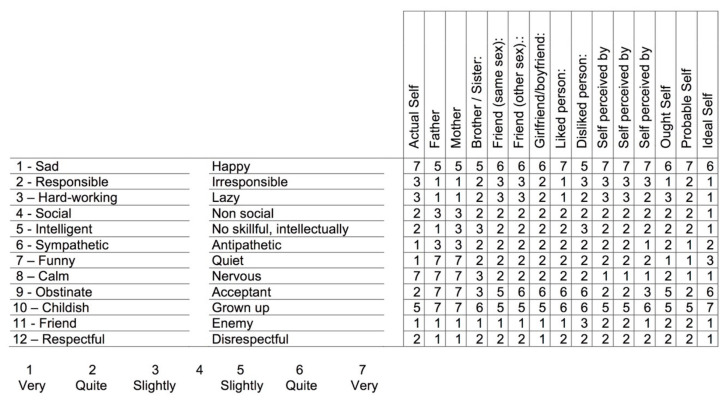
Repertory grid as completed by a 17-year old adolescent male. Elements in columns and constructs in rows; both are elicited from the participant during the interview. Ratings from 1 to 3 indicate allocation of a given element in the left pole of the construct, while ratings from 5 to 7 indicate allocation in the right pole (middle point is 4).

To assess the personal construct systems, Kelly [6] conceived the repertory grid technique (RGT), a structured procedure similar to an interview (see [24,25]). This tool eloquently illustrates PCP’s perspective on self-knowledge. First, interviewees name a sample of significant others and self-elements (e.g., most frequently Actual Self and Ideal Self, but other self-elements can be considered, like “Ought Self” or “Self as perceived by particular others”), then they are asked to identify similarities and differences between them; in this way, a sample of bipolar constructs, like “sad–happy”, is elicited. Finally, individuals rate every self and other element in each of the elicited constructs (usually on a 7-point Likert scale). At the end, this technique provides a qualitative and quantitative data matrix (see Figure 1) that allows the computing of several cognitive structure measures, such as differentiation, polarization, discrepancies between Actual Self, Ideal Self, and Others (as perceived by the interviewee), as well as several measures of psychological conflict [24,25,26].

Compared to more widely used instruments, the RGT provides idiographic self-knowledge (concerning content and organization patterns) and allows qualitative and quantitative standardized measures, a recommended combination to assess self-knowledge organization [12,13,14].

Finally, previous studies have revealed the RGT as a promising technique for understanding several psychological aspects central to adolescents’ self-knowledge development, which will be reviewed in the second part of the next two sections, preceded by a summary of contributions from developmental approaches.

### 1.2. Differentiation and Conflicts in the Organization of Self-Knowledge in Adolescence

An influential and comprehensive perspective on the development of the self in adolescence is offered by S. Harter ([5], for a review). According to her perspective, important cognitive changes occur in adolescence that, combined with new social contexts, promote reorganizations of self-knowledge. S. Harter describes a detailed developmental trajectory comprising three phases across adolescence. During early adolescence, childhood self-representations give place to an increasing number of single self-descriptive abstractions. Adolescents personal characteristics increasingly differentiate in terms of relational contexts (e.g., me-with-my-father, me-with-my-best-friend). Similarly, early adolescents distinguish their competence in an increasing number of different life domains (e.g., scholastic competence, social competence, physical appearance, behavioral conduct, close friendship, romantic appeal, job competence). At the same time, self-esteem becomes more differentiated according to different social contexts. By middle adolescence, adolescents perceive contradictions among the differentiated personal characteristics; thus, they experience psychological conflicts and preoccupations about the existence of false selves and the identification of their true self (e.g., “talkative” or “secretive”?) [5,10]. Later, those attributes formerly perceived as contradictory can be combined into a more coherent self-picture (e.g., “Basically, I am flexible: I am talkative with friends and secretive with my parents”). Research suggests that older adolescents are often able to organize their self-knowledge in this integrative way, provided they can count on the appropriate support (e.g., a more mature person) [5,27]. Other studies suggest that some kinds of conflicts diminish at late adolescence, but others do not [28]. Also, older adolescents become capable of a more nuanced and less polarized view of themselves and others. Indeed, external support seems to have an important role in the adolescent task of organizing self-knowledge. The developmental sequence described by S. Harter [5,29] is particularly evident when adolescents receive some support in the task of organizing self-knowledge (e.g., identifying conflicts); when support is not available, adolescents show less cognitive sophistication and developmental advances seem to be slower and more linear [27].

According to the PCP’s framework, self-knowledge differentiation and hierarchical integration increase as life goes on, and adolescence should not be an exception. Considering that the RGT has been fruitful in providing measures of differentiation, integration, and conflicts, in this study, we aim to measure two of the most consolidated aspects of the overall cognitive structure (and its sophistication), namely, global differentiation and polarization [30]. Differentiation refers to the diversity of different dimensions of meaning that a person has available to anticipate interpersonal and personal events. Polarization refers to rigid (black and white) knowledge about self and others along the dimensions of meaning, as opposed to the ability to build up a nuanced perspective.

Reviewing studies about developmental change in children and early adolescents, Jablonsky and Lester [23] suggested the predicted developmental trend of increasing differentiation and integration. Also, they mentioned an increase in the number of constructs and the constructs becoming more abstract and less concrete (also [28]). However, only a few developmental studies (cross-sectional or longitudinal) have addressed this issue at different stages of adolescence. Zhang et al. [14] found no correlation between differentiation and age across adolescence in a sample of Chinese adolescents. However, they attributed this result to the nomothetic approach they used (the constructs were provided by the researchers). Other studies measured differentiation and polarization in adolescence but included adolescents of only one age group [31,32].

Yet, in a system where constructs are considered to be interconnected, it is possible that contradictions arise between constructs and imperfections happen in the integration of the system, like fragmentation and conflicts. The RGT has been fruitful in providing measures of intrapersonal conflict (for a review see, for instance, [26]). In a cross-sequential study involving participants from 6 to 18 years old, Oosterwegel and Oppenheimer [28,33] conceived a composite measure of conflicts within (e.g., Ideal Self from perceived parents’ perspective) and between self-concepts (e.g., Ideal Self and Actual Self from own perspective), partially based on RGT data. They found that the number of conflicts within self-concepts (e.g., Actual Self from adolescent’s own standpoint) peaked between 12 and 14 years old and stabilized in subsequent years [33]. Concerning conflicts between self-concepts, they found different pathways. The conflicts between Actual and Ideal Selves from the perceived perspective of parents increased throughout childhood, stabilized during adolescence, and tended to decrease slightly in late adolescence. However, they found no age-related changes in the conflicts between other self-concepts, such as the conflict involving Actual and Ideal Selves from the youth perspective [28]. In sum, they found that different conflicts showed different developmental pathways across childhood and adolescence, some possibly conforming to the developmental sequence proposed by S. Harter [5], but others not.

However, in Oosterwegel and Oppenheimer studies, as in S. Harter’s [29], the identification of conflicts strongly relies on adolescents’ awareness, and a certain amount of support (especially in S. Harter’s research) was provided for adolescents to relate their self-descriptions. Conversely, RGT offers the possibility of computing several measures of conflict that do not require the awareness of the person nor explicit cognitive activity in identifying conflicts (as most likely happens when adolescents live their daily lives).

In this study, we focus on implicative dilemmas [26], a particular kind of conflict identified in repertory grids (described in detail in the Methods section). Briefly, implicative dilemmas involve the Actual and Ideal Selves as they are perceived by two distinct personal constructs that are linked. This is a conflict in which a desired change in one construct is blocked because it implies an undesired change in another attribute. Research has shown that this type of conflict is related to psychological distress [34,35], including in adolescents [31]. However, no previous studies have focused on developmental differences in this kind of conflict.

### 1.3. Self-Discrepancies and Identity Construing in Adolescence

In the developmental literature, another piece of evidence about cognitive differentiation refers to the increasing distance between the Actual Self and the Ideal Self throughout adolescence [36]. This Actual–Ideal self-differentiation (A–I; also called self-disparity or self-discrepancy by different authors), similar to other kinds of self-differentiation, has been associated with different, sometimes contradictory, psychological meanings. For instance, the increase of self-discrepancies with age has been conceived as part of the internalization of self-guides taking place during adolescence [37,38]. During this process, external self-evaluation and self-regulation standards provided by significant others (as models, or as young people perceive others’ expectations to the self) give place to adolescents’ own standards and to a more autonomous, independent self-regulation. In this context, the research on self-discrepancies suggests that the Ideal Self of adolescents seems to become progressively more differentiated from the significant others (as they are perceived by the adolescent), from the Ideal Self he/she perceives others have for him/her, and from the self-perceived Actual Self, as they age [5,36,37,38,39,40].

In addition, the A–I differentiation has been considered by some authors to be associated with self-esteem, an association especially important in the context of the adolescents’ development [5,41]. According to this perspective, self-esteem emerges from the distance between the actual attributes one perceives about him or herself and those which he/she aspires to possess. Some evidence supports this association between A–I and global self-esteem, as measured by traditional standardized scales [40,42], especially when idiographic measures of Actual and Ideal Self attributes are considered [43]. However, evidence about changes in global self-esteem from childhood on is not consensual and is not always consistent with the developmental increase proposed to A–I throughout adolescence. Some studies suggest that global self-esteem diminishes throughout adolescence to start increasing in emerging adulthood [44,45], whilst others propose that it diminishes only in early adolescence and increases in the following years [46,47].

The research on individuation and identity formation is another important contribution to the field [3,48]. Adolescents are expected to become progressively more independent from parents, to define autonomous relationships with the world and to establish themselves as a singular constellation of attributes. Accordingly, there is evidence that a sense of being different from others increases across adolescence [49,50,51,52]. However, in the end, as young people go through emerging adulthood, the role of integrative processes seems to be of great importance in the task of combining a diversity of psychosocial possibilities in a unique, committed identity [3,50].

In the PCP field, the topics of identity and self-discrepancies are linked. Different authors have proposed, based on RGT, that the construing of self-identity relies on the degree of similarity between the self-perceived Actual Self, how significant others are perceived, and the Ideal Self [24,53]. First, the discrepancy between the Actual Self and significant others (as they are perceived by the adolescent) (i.e., A–O) is considered a measure of identification with others, or distinctiveness, an important component of the classic view of identity [52]. Adams-Webber (see, for example, [54]) argued that people tend to perceive similarities between themselves and others in about 62% of characteristics and that this balance is achieved in adolescence. From his point of view, the supremacy of characteristics shared with others (as opposed to those that are distinct) serves the function of better differentiating self and others, such as a figure (the differences) against a background (the similarities). Some developmental research within the scope of PCP shows, for example, that children (8–11 years) perceive themselves to differ from their parents less than adolescents (see [54]), and that this differentiation increases throughout adolescence. Yet adolescent efforts to maximize this distinction can compromise the validation of the self [55].

Second, the differentiation between Actual Self and Ideal Self can be understood as a degree of acceptance of the self and/or as a predictor of self-esteem [56]. A high discrepancy promotes invalidation of the self, unpleasant effects, and a need to change [55]. Results regarding the A–I developmental pathway are inconclusive, as some studies showed no changes across childhood and adolescence [28] and others suggested a peak in middle adolescence [55]. Less studied, the differentiation between Ideal Self and Others (I–O) refers to the appreciation that one has about Others as more or less adequate [24] or as potential role models, thus inspiring the direction of personal change [55]. This distance increases across adolescence, and greater distances would hinder the social validation of the adolescent’s self [55].

Overall, these three self-discrepancies have been studied more in relation to psychological adjustment than to cognitive development. Previous studies on adults suggest that greater differentiations are related to psychological distress [56]. A study on adolescents found a relation with depressive syndrome only for the A–I differentiation [31]. In addition, with the inclusion of the Ideal Self and the anticipation of a possible (maybe future) self it represents, PCP somehow addresses another important component of identity, the sense of personal continuity [52].

### 1.4. The Current Study

In summary, different developmental approaches suggest an increase of self-knowledge differentiation across adolescence, with very few studies highlighting a peak of self-perceived conflicts within self-knowledge by middle to late adolescence. These conflicts could possibly be reorganized in a more coherent, integrative way by late adolescence or emerging adulthood since adolescents are provided with support. In addition, intrapersonal conflicts seem to be understudied despite the numerous theories addressing its role in development and psychological adaptation (for some exceptions, see [5,27,28,57]). In our view, PCP could offer an integrative approach, providing potential insights into the understanding of self-knowledge development during adolescence, thus addressing a gap in the current literature.

The aim of this cross-sectional study is to explore the self-knowledge organization in adolescence with the help of PCP’s conceptual and methodological contributions when considering predictions from developmental perspectives. Among PCP’s conceptual contributions, we highlight the entanglement of knowledge about the self and knowledge about significant others or its ability to understand personal change in the face of the experience. Examples of methodological contributions are a set of structural, idiographic measures derived from RGT, including conflicts, which may reveal cognitive structures that people are not immediately aware of.

As mentioned above, the administration of the RGT does not provide support to adolescents in the particular task of relating self-attributes. Hence, developmental differences are expected to be slow to appear; for instance, the emergence of conflicts and, especially, their subsequent resolution, would be expected to occur later than proposed by S. Harter’s developmental sequence [5,10,27]. With this in mind, this study compares two periods of adolescence, namely, early and late adolescence.

Our central hypothesis anticipates that self-knowledge organization will be different in early and late adolescents, such that the older sample will show (a) higher global differentiation; (b) lower polarization; (c) more participants with intrapersonal conflicts; and (d) higher discrepancies between Actual Self, Ideal Self, and Others.

## 2. Materials and Methods

### 2.1. Participants

A total of 68 participants completed this study. All participants were students from six Portuguese middle and secondary schools. Thirty-three 7th graders (25 females) integrated the early adolescence sample (*M*_age_ = 12.24, *SD* = 0.50), and 35 (22 females) 12th graders the late adolescence sample (*M*_age_ = 17.29, *SD* = 0.79). More girls (69.12%) than boys participated in both samples, Adolescence Phase × Gender, χ^2^ = 1.324, *p* = 0.250. All late adolescence participants reported their will to apply to university studies, whereas 7 early adolescence participants planned not to do it.

### 2.2. Instruments and Measures

The RGT [6] was used to obtain six self-knowledge measures from each participant. Repertory grids were based on 15 elements: Actual Self, Father, Mother, Brother or Sister (the closest in age), Friend (same gender), Friend (other gender), Boyfriend or Girlfriend (or romantic interest), Liked Person, Disliked Person, Self as perceived by father, Self as perceived by mother, Self as perceived by best friend, Ought Self (how one thinks he/she has the obligation or duty to be), Probable Self (in 1 year), and Ideal Self. Twelve constructs were elicited using the dyadic method: participants were asked to compare a pair of elements (e.g., Actual Self and Father) first searching for similarities (e.g., “which characteristics have these two people in common?”, then asking the opposite of that characteristic) and then for differences (e.g., “and in which characteristics do they differ?”). Finally, participants used a 7-point Likert scale to rate every element in each construct: 1—very, 2—quite, and 3—slightly, related to the first pole of the construct (e.g., sad); 4—middle point; and 5—slightly, 6—quite, and 7—very, related to the other pole (e.g., happy). Every individual data matrix was processed by the GRIDCOR v.4.0 software program [24], which computed the following measures (more details about the psychometric properties of the repertory grid and its measures in [24,25,30]).

#### 2.2.1. Global Differentiation

The percentage of variance accounted for by the first factor provided by the correspondence analysis of each repertory grid data matrix identifies the most important dimension of meaning, and its size is considered to be a measure of global differentiation [30]. A low value indicates a higher differentiation and a multidimensional way of interpreting self and others, whereas a high value indicates a more unidimensional construing (i.e., this factor alone tends to explain much of the variance). Test–retest reliability with adult samples revealed correlations of 0.61, 0.72, and 0.67 at one hour, one week, and one month later, respectively [58], and other studies found higher correlations [59,60]. Providing support for validity, this measure showed correlations with other established measures of differentiation obtained from repertory grids ranging from 0.83 to 0.95 [30,60]. In a recent study on late adolescents, a subclinical depression subsample showed a lower value (higher differentiation) (*M* = 42.01, *SD* = 8.55) compared to a sample with no symptoms (*M* = 49.72, *SD* = 8.35) [31]. First-year students in university increased their global differentiation at the end of the academic year (first: *M* = 48.24, *SD* = 13.23; after: *M* = 46.12, *SD* = 12.69). An adult sample in Feixas et al.’s study [56] obtained a mean value of 42.81 (*SD* = 9.92).

#### 2.2.2. Polarization

This is the percentage of extreme rates (1 and 7) in the grid and is considered to be a measure of the “all or nothing” thinking. A high value is considered to reveal a rigid construing and a low value a more nuanced construing. The abovementioned study by Feixas et al. [58] found a test–retest reliability with correlations of 0.89, 0.83, and 0.71, at one hour, one week, and one month later, respectively. Caputi and Keynes [59] found correlations from 0.84 to 0.92, with re-test at one and two weeks. Feixas et al. [30] showed low correlations (from 0.08 to 0.14) with different measures of differentiation derived from repertory grids, attesting to the discriminant validity of polarization. In a study mentioned earlier [31], late adolescents with subclinical depression (*M* = 26.01, *SD* = 17.55) and with no-symptoms (*M* = 32.35, *SD* = 11.74) showed similar values of polarization. An adult sample in Feixas et al.’s study [56] obtained a mean value of 25.11 (*SD* = 13.34) for polarization.

#### 2.2.3. Presence of Implicative Dilemmas

**Figure 2:**
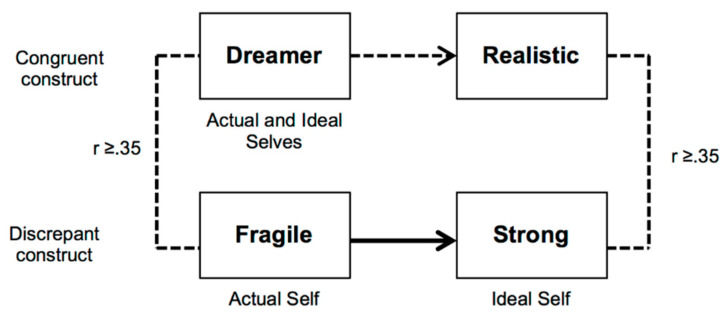
Illustration of an implicative dilemma found in a repertory grid completed by an adolescent.

This is an intrapersonal conflict that is identified in repertory grids by a correlation between a congruent construct (Actual and Ideal Selves are perceived as alike, rated difference no higher than 1 point) and a discrepant construct (Actual and Ideal Selves are perceived as distinct, score difference higher than 3 points). A cutoff of r > 0.34 is used to include only correlations of, at least, medium effect size [61]. This specific between-constructs correlation is considered indicative of an implicative dilemma expressing that the desired change revealed by the discrepant construct is associated with a change in direction to the undesired pole of the congruent construct. Figure 2 shows an implicative dilemma identified in a grid from our study. The adolescent sees himself as “fragile” but he wishes to become “strong” (as indicated by his rating of the Ideal Self). He also considers himself a “dreamer”, and in that he meets his Ideal self. The dilemma emerges because the desired change in the direction of becoming “strong” implies, in his construct system, to become more “realistic”, which invalidates a positive attribute of himself (being a “dreamer”). This is a kind of conflict that possibly blocks or limits desired personal development. Several studies show a higher prevalence of implicative dilemmas in a diversity of clinical compared to community adult samples (for a review see [35]). However, in spite of a higher prevalence in clinical adult samples (53% of the subjects show at least one implicative dilemma), implicative dilemmas are quite present (34%) in non-clinical samples [26]. A study with university students found that implicative dilemmas were present in 53.57% of the participants at the start of their first year, and in 32.14% at the end of that year [62]. A recent study revealed a higher presence of implicative dilemmas in depressed late adolescents (74%) compared to their no symptomatic counterparts (25%) [31].

#### 2.2.4. Discrepancies between Actual Self, Ideal Self, and Others

The mean value of the Euclidian distances between the scores of the Actual Self, Ideal Self, and Others (Father, Mother, Brother/Sister, Friend/same gender, Friend/other gender, Boyfriend/Girlfriend, Liked Person, and Disliked Person) provided three self-discrepancy measures, A–I, A–O, and I–O. All three discrepancies have been interpreted as measures of cognitive differentiation [28,36], such that greater distances correspond to higher differentiations. In addition, each discrepancy has been considered to measure different psychological constructs. The A–I has been interpreted as an approximation to self-esteem [24,63], in that greater distances might indicate lower self-esteem. The A–O has been considered as a measure of the identification with others [24,55,63], going from a self-perception of social isolation of the self (higher distance) to the identification with others (lower distance). Finally, the I–O has been seen as a measure of the perceived adequacy of the Others, going from a positive perception (lesser distance) to a perception of Others as inadequate (greater distance) [56], or a measure of how much the Ideal mirrors (lesser distance) or is different (greater distance) from Others [55].

Several studies have reported the test–retest reliability for self-discrepancies. For instance, Feixas and colleagues [58] found correlations of 0.92, 0.88, and 0.78, for A–I, and 0.94, 0.89, and 0.85 for A–O, for re-test at one hour, one week, and one month later, respectively. Caputi and Keynes [59] found correlations ranging from 0.61 to 0.81 with re-test at one and two weeks. All self-discrepancies were higher for adults with depression in studies comparing them with a non-clinical sample [56].

In their study with late adolescents, Carapeto and Feixas [31] found that mean values of A–I were higher in the sub-clinically depressed sub-sample (*M* = 0.51, *SD* = 0.25) than in the no-symptoms sub-sample (*M* = 0.29, *SD* = 0.28); however, A–O mean values were similar in the two sub-samples (*M* = 0.46, *SD* = 0.19 vs. *M* = 0.40, *SD* = 0.16 for those with no symptoms), as were I–O values (*M* = 0.40, *SD* = 0.14 vs. *M* = 0.37, *SD* = 0.13 for those with no symptoms).

### 2.3. Procedure

This research project was approved by the Consejo de Departamento de Personalidad, Evaluación y Tratamiento Psicológicos, University of Barcelona. The study was conducted in accordance with the ethical standards of the American Psychological Association. The informed consent to participate in this study was obtained from the school principal, parents, and adolescents, prior to beginning data collection.

The RGT was administered to groups of up to 8 adolescents in a school classroom outside of the class schedule. The data analysis was performed with the help of two computer programs. First, the GRIDCOR v. 4.0 [24] was used to analyze the data from every individual’s repertory grid and to compute the measures of self-structure for each participant. Then, these values were entered into SPSS v.24 to perform statistical comparisons between the early- and late-adolescence samples.

The similarity to the normal distribution was checked for the quantitative variables (Global Differentiation, Polarization, A–I, A–O, and I–O) by phase of adolescence (Kolmogorov–Smirnov), as well the homogeneity of variances (Levene’s tests). A multivariate analysis of variance (MANOVA) was conducted to explore possible global differences in self-knowledge organization between early and late adolescents. These five measures were entered as dependent variables, while phase of adolescence (early and late) was introduced as a factor. As the global effect was found significant, a series of one-way ANOVAs were performed as post-hoc tests to appreciate the effects of phase of adolescence (early and late) on each quantitative measure. Chi-square was used to test the association of the presence of implicative dilemmas with phase of adolescence. Effect size (ES) measures (η^2^ and φ) and observed powers (computed using α = 0.05) were computed as well. A difference or an association was considered to achieve statistical significance when *p* < 0.05; marginal significance (*p* < 0.10) was also reported.

## 3. Results

The MANOVA results revealed that the organization of self-knowledge (considering the set of the five quantitative measures together) is significantly different in early and late adolescents, F (5, 62) = 3.202, *p* = 0.012; Wilk’s lambda = 0.795, η^2^_p_ = 0.205, observed power = 0.857. Table 1 shows mean values for each quantitative measure of self-knowledge organization and the percentage of adolescents presenting at least one implicative dilemma, by phase of adolescence, as well as ANOVA and chi-square statistics.

Polarization is lower and the Actual–Ideal self-discrepancy tends to be higher for late adolescents. No differences were found between early and late adolescents concerning global differentiation, and the Actual–Others and Ideal–Others discrepancies. A tendency (small to medium ES) was found for conflicts (implicative dilemmas) to be more frequent in participants of late than those of early adolescence.

## 4. Discussion

The aim of this study was to explore the potential of Kelly’s PCP and the RGT for assessing a diversity of self-knowledge organization aspects which have been investigated in the developmental literature (including conflicts, seldom receiving empirical attention) and to examine whether the RGT could be sensitive to differences possibly related to development in adolescence. The hypothesis anticipated differences between self-knowledge organization in early and late adolescence. Specifically, higher global differentiation, higher self-discrepancies, and lower polarization in late (compared to early) adolescence, as well as a higher number of participants with conflicts were predicted. Considering this set of self-structure variables together, we can say that our late adolescent sub-sample was different from our sub-sample of early adolescence, with a large effect size. However, these effects varied across the self-knowledge variables studied. Later adolescents exhibited lower polarization and a tendency to higher Actual–Ideal self-discrepancy and higher likelihood of intrapersonal conflicts with small to medium effect sizes. In contrast, no significant differences were found between early and late adolescents’ self-knowledge in terms of global differentiation nor in the self-others discrepancies (Actual Self–Others and Ideal Self–Others).

### 4.1. Global Differentiation, Conflicts, and Polarization

The results of the global differentiation of self-knowledge suggest that younger and older adolescents do not differ significantly in the amount of dimensions of meaning available to make sense of themselves and others. This result is consistent with other studies that found no developmental changes in differentiation (e.g., [14,64]), but does not support the assumptions of increasing differentiation throughout adolescence proposed by the cognitive-developmental approach [5,27]. More research is needed to clarify the developmental trajectories of global differentiation using the RGT, including studies with larger samples that could also consider the possible role of other variables. For instance, several studies showed that girls differentiate their self-knowledge more than boys [5,27,63,64] and others indicate the relevance of the experience of stressful events [6,10,64].

Turning to another aspect of the organization of self-knowledge, polarization decreased in late adolescence in the expected direction, thus suggesting a higher cognitive sophistication in the older adolescents [5,36]. Although late adolescents construe the personal and interpersonal reality with a number of dimensions of meaning which is similar to their younger counterparts, they apply them using a larger variety of shades. In addition, a longer developmental pathway of decreasing in polarization is suggested if the adult sample from Feixas and colleagues’ study [56] is considered, such that early adolescents polarize their self-knowledge more than late adolescents, who are still more polarized than adults (*M* = 25.11, *SD* = 13.34).

Somehow in the expected direction, results showed implicative dilemmas presence in about two-thirds of older, but only in a grossly third of younger adolescents. Additionally, the comparison of our adolescent samples with Spanish adult samples suggests that the presence of conflicts peaks in late adolescence (60% of the older adolescents showed at least one implicative dilemma, against 39% of the youngers), and is lower in adulthood (for instance, 36% in [65]). This pattern looks similar to the developmental sequence proposed by S. Harter [5,29]. It is possible that many late adolescents of the present study developed conflictive relationships among their differentiated constructs but without enough integrative skills to solve these conflicts in an autonomous way (as most adults could do). This apparent delay in resolving implicative dilemmas (compared to the timing of final adolescence proposed by S. Harter) supports research showing that some types of conflicts do not diminish in late adolescence [28,33]. On the other hand, it recalls the distinction between functional and optimal cognitive performance proposed by dynamic skills theory [10] and favors the idea that implicative dilemmas may speak of a conflict structure that people construe out of immediate awareness at functional cognitive levels, and is thus more frequently resolved in the more mature years of adulthood.

From this developmental perspective, these conflicts are both outcomes of cognitive advances and challenges to psychological adjustment [5,10]. Currently, some literature links the presence of internal conflicts to psychological difficulties [3,5,57], and the recent research on implicative dilemmas in adolescents [31] and adults [35] goes in the same direction. A meta-analysis research by Gray and colleagues [34] found an association of goal conflict (including implicative dilemmas) with well-being, which is stronger concerning distress than positive outcomes. However, there is a possibility that the psychological discomfort produced by the conflicts can promote efforts to achieve a more integrated, coherent self-knowledge, and, by doing so, intrapersonal conflicts can have an important role in the promotion of self-knowledge development. In this context, S. Harter [5] (also [27]) stressed the importance of social support by a mature person in the task of combining and integrating conflicting self-attributes. In the particular case of implicative dilemmas, some studies suggest that they can be solved in the psychotherapy context, along with a decrease of severity in psychopathological symptoms [66]. More research is needed, especially longitudinal studies, to understand the developmental aspects involved in the formation and resolution of this kind of conflict, as well as its role in psychological (mal)adaptation.

### 4.2. Self-Discrepancies and Identity Construing

The prediction of a higher differentiation in late adolescence is supported by marginally higher A–I discrepancy. As the A–O and I–O discrepancies did not follow the same pattern, the general cognitive development explanation [5,10] seems to be insufficient to account for the results. Other explanations, more concerned with the psychosocial adaptation dynamics in adolescence, could be considered. For instance, the late adolescents’ higher A–I discrepancy could be related to advances in the process of ideal self-guides internalization [38,67], to the setting of more ambitious personal goals which could maximize the motivation to take personal actions in the desired directions [38,40,67,68], or to promote the conditions to essaying new roles, as expected from a psychosocial exploration phase in adolescence [3,50,55,69].

Nevertheless, the similar A–O does not support the hypothesis and suggests that early and late adolescents identify themselves with others to the same extent. This finding does not support the expected advances in the processes of individuation and personal identity formation [3,48,50,51] and diverge from the results of Strachan and Jones [55], which suggest an increase throughout adolescence in a study that includes only the distance to parents. Likewise, the I–O discrepancy did not support previous research [55], suggesting that the perceived adequacy of significant others was similar for younger and older adolescents, as well the inspirer role of those others in the construing of personal goals. Some methodological aspects may contribute to our discordant results, such as the composition of the “others”, which, in the present study, group together a diversity of meaningful people (parents, siblings, friends, liked and disliked people) while other studies have focused on the knowledge about particular others [70], mainly parents [54,55]. At this light, the results concur with S. Harter’s [5] proposal that the influence of significant others on the building of self remains throughout adolescence, although different others (e.g., peers or parents) may play a more prominent role in particular stages or domains of self-definition (e.g., scholar or social). Future studies could disentangle this knowledge about particular others and their position in relation to the Actual and the Ideal selves.

High self-discrepancies, such as A–I, have also been considered to challenge psychological adjustment. The higher A–I discrepancy found for late adolescents may suggest a lower self-esteem, which is consistent with existing studies on global self-esteem [44], and contradicts others [46]. Thus, the relationship between A–I discrepancy and self-esteem deserves further research. In addition, some studies have linked depressive affect to higher A–I discrepancy in adolescence (e.g., [31,39,42,67,71,72]), as other studies found that this relationship is stronger for those exhibiting more sophisticated cognitive abilities [73]. Besides, our results are consistent with research reporting the increase in the incidence of depression in girls during adolescence (e.g., [74,75,76]), since females are more numerous than males in our sample. The possibility that a high level of discrepancy could be experienced as internalized maladjustment by many late adolescents [40,42] deserves further research, considering the possible risk of future maladjustment as well. For instance, some research signals the risk that A–I discrepancy could stabilize at high levels at the end of adolescence [38], and that adolescents’ associated low self-esteem could open a pathway to depression in adulthood [77,78].

### 4.3. Limitations of the Study and Implications for Future Research

Some methodological aspects of this study can limit the developmental interpretations and generalizations of the results, namely, the small size of samples, the unique context of participants’ recruitment (i.e., public schools, regular programs), the particular characteristics of those who volunteer to participate in a psychology study out of class time, or the cross-sectional design of the study. With these limitations in mind, our discussion of the results has remained cautious and, rather than definitive conclusions, a set of alternative explanations and questions for future research have been presented. In synthesis, this study stresses the relevance of further longitudinal research (or at least cross-sectional samples representing the different stages throughout adolescence) with sizeable samples of both gender adolescents living a diversity of scholar and vocational pathways or living with different mental health conditions (including clinical samples), that could enlighten the pathways of diverse aspects of self-knowledge organization, emotions, and other daily experiences, in the making of psychological (mal)adaptation. Thus, some of the variables to be included would be daily life events and stressors, self-esteem (for which a high correlation with the A–I discrepancy grid measure used in this study would be expected), affects, and psychopathology symptoms (internalized and externalized), as well as self-regulation and socioeconomic status [79].

## 5. Conclusions

In spite of the already mentioned methodological limitations of this study, our results encourage some working conclusions that need further research. A reasonable conclusion is that PCP seems to offer a conceptual and methodological framework facilitating a comprehensive understanding of the self-knowledge development dynamics in adolescence. In particular, the RGT showed to be a sensitive instrument to variations in several aspects of the self-knowledge organization possibly related to developmental changes. In addition, it offers new possibilities of measuring and understanding intrapersonal conflicts and its role in psychological adaptation. Relatedly, besides research, the theoretical and methodological framework of PCP has provided fruitful ways to approach clinical and educational practices (e.g., [15,80]). Also, this study stresses the interest of exploring not only the cognitive-normative advances, but also the dynamics of self-knowledge organization in the context of the adolescents’ daily experiences (e.g., developmental tasks, adversities, emotions) in construing self and others, and the possible vulnerabilities that emerge too. We hope that clarifying the role of these variables in the reorganization of self-knowledge and its role in the making of psychological adaptation would help us to prevent the possible vulnerabilities in adolescence from being more than transitory.

## Figures and Tables

**Table 1:** Descriptive statistics and statistical contrasts for measures of self-knowledge by phase of adolescence.

	Mean (SD)		*F* (1, 66)	*p*	η^2^_p_	Observed Power
	Early (*n* = 33)	Late (*n* = 35)				
Differentiation	45.66 (9.98)	43.56 (8.15)	0.906	0.345	0.014	0.155
Polarization	40.35 (19.75)	29.42 (14.85)	6.695	0.012	0.092	0.722
Actual–Ideal	0.31 (0.25)	0.43 (0.24)	3.985	0.050	0.057	0.503
Actual–Others	0.38 (0.22)	0.43 (0.16)	1.010	0.319	0.015	0.168
Ideal–Others	0.43 (0.18)	0.38 (0.14)	1.736	0.192	0.026	0.255
	Number of cases (%)		χ^2^ (1, 68)	*p*	φ	
Dilemmas	13 (39.4%)	21 (60%)	2.885	0.089	0.206	

*Note.* Early—early adolescence; Late—late adolescence; Actual–Ideal—discrepancy between Actual and Ideal Selves; Actual–Others—discrepancy between Actual Self and Others; Ideal–Others—discrepancy between Ideal Self and Others.
